# Detection of Drowsiness among Drivers Using Novel Deep Convolutional Neural Network Model

**DOI:** 10.3390/s23218741

**Published:** 2023-10-26

**Authors:** Fiaz Majeed, Umair Shafique, Mejdl Safran, Sultan Alfarhood, Imran Ashraf

**Affiliations:** 1 Department of Information Technology, University of Gujrat, Gujrat 50700, Pakistan; fiaz.majeed@uog.edu.pk (F.M.); umairg92@gmail.com (U.S.); 2 Department of Computer Science, College of Computer and Information Sciences, King Saud University, P.O. Box 51178, Riyadh 11543, Saudi Arabia; mejdl@ksu.edu.sa; 3 Department of Information and Communication Engineering, Yeungnam University, Gyeongsan 38541, Republic of Korea

**Keywords:** advanced driver assistance systems, deep learning, drowsiness detection, neural network, road safety

## Abstract

Detecting drowsiness among drivers is critical for ensuring road safety and preventing accidents caused by drowsy or fatigued driving. Research on yawn detection among drivers has great significance in improving traffic safety. Although various studies have taken place where deep learning-based approaches are being proposed, there is still room for improvement to develop better and more accurate drowsiness detection systems using behavioral features such as mouth and eye movement. This study proposes a deep neural network architecture for drowsiness detection employing a convolutional neural network (CNN) for driver drowsiness detection. Experiments involve using the DLIB library to locate key facial points to calculate the mouth aspect ratio (MAR). To compensate for the small dataset, data augmentation is performed for the ‘yawning’ and ‘no_yawning’ classes. Models are trained and tested involving the original and augmented dataset to analyze the impact on model performance. Experimental results demonstrate that the proposed CNN model achieves an average accuracy of 96.69%. Performance comparison with existing state-of-the-art approaches shows better performance of the proposed model.

## 1. Introduction

Transportation is an essential part of human lives and a significant portion of a country’s economy comes from the transportation industry. While being a source of safe and fast travel, lack of driver vigilance, fatigue, and drowsiness may lead to accidents involving injuries and fatalities [1]. Driver drowsiness is responsible for a large number of accidents in the world. Different studies report 20% to 50% of accidents are related to driver fatigue and drowsiness on certain roads [2,3]. Drowsiness is when someone feels dizzy or experiences involuntary sleep, primarily due to a lack of sleep or mental or physical fatigue. It can be particularly hazardous when there is a need for a consistently high level of attention, such as in industrial work, mining, and driving to avoid unwanted and life-threatening events [4,5]. When it comes to drowsy driving, it has severe implications for road safety. Along with other contributing factors such as speeding, drinking, and driving, and not wearing seat belts or helmets, drowsy or fatigued driving is also considered a major source of road accidents [6,7].

Over the last few years, physical and life losses because of road accidents have increased. According to reports on road safety [8], out of 1.35 million mortalities, approximately 37% of people die yearly due to drivers’ drowsiness in road accidents. Overall, it is the eighth leading cause of death and ranked the first cause of mortalities for people aged between 5 and 29. Taking energy drinks, coffee, or stopping to take a short nap while traveling for a long time helps drivers stay alert, but the effect remains for the short term [9]. Moreover, these remedies may only be effective when a driver is aware of fatigue [10].

### 1.1. Research Objectives

Keeping with the above discussion, a driver’s acts are critical to road security, both for the driver and other people traveling on roads. Due to its significant importance in saving lives, driver drowsiness detection has received increased attention lately. Several studies are found in the literature that focus on detecting different levels of alertness in drivers using unique facial cues such as head poses, eye movement, and other facial expressions [11,12]. While ongoing research shows promising advancements, core challenges, such as accurate and real-time drowsiness detection, still need to be addressed. Current driver yawning detection systems are either expensive or need more robustness [13,14].

Drowsiness among drivers causes injuries and sometimes the death of millions of people annually; there is a need to develop a system with high accuracy, precision, and robustness. Detection of drowsiness is a critical factor for successfully preventing road accidents. The objective of this research is to propose a deep neural network model that performs relatively better in terms of accuracy and other measures.

### 1.2. Research Contributions

This study aims to propose a more accurate driver drowsiness detection method to reduce road accidents. The following are the major contributions of this study:

- A deep convolutional neural network (CNN) is designed in this study for driver drowsiness detection. The model is optimized regarding the number of layers, neurons in each layer, etc. In addition, a hybrid deep learning model CNN-RNN (recurrent neural network) that combines CNN and RNN deep learning models is also used.
- Experiments involve using the publicly available YawDD dataset. To reduce the impact of smaller datasets, data augmentation is used. Separate experiments are performed using augmented and original datasets for performance comparison.
- For model training, facial features like the mouth aspect ratio (MAR) are used, which are extracted using the Dlib library. The effectiveness of the proposed model is evaluated using multiple performance metrics such as precision, recall, and F1 score. Moreover, performance comparison with existing state-of-the-art models is also carried out.

The remaining part of this study is organized as follows. Section 2 presents the literature review on driver drowsiness detection. Section 3 describes the methodology, while experimental results are explained in Section 4. Lastly, Section 5 presents the conclusion and future recommendations.

## 2. Literature Review

Driver drowsiness detection techniques fall into three main categories. The first category is the biological feature technique that involves analyzing physiological signals [15], skin temperature, and galvanic skin response (GSR) to measure physical conditions that change with the level of drowsiness or fatigue [16,17,18,19]. The second category is vehicle movement indicator techniques specifically focused on driving applications to detect abnormal driving behavior due to fatigue or drowsiness, such as random braking, lane positioning, abnormal speeding, and abnormal steering. We can observe these kinds of behavior with the help of different sensors in the vehicle [2,19,20]. Vehicle movement indicator techniques have several restrictions such as road shape, vehicle type, driver expertise, and the situation, and more importantly, it needs more time to acquire all these parameters [14,21]. Both categories are invasive, requiring extra equipment or sensors to detect drowsiness [13]. The stated limitation makes both techniques inappropriate to implement in real-time. Consequently, most studies focus on the third category, which uses the behavioral features of drivers.

The behavioral feature technique is noninvasive and involves computer vision for drowsiness detection. For real-time visual analysis of behavioral features, a camera is needed [11,12]. Behavioral measures such as unusual eye movement, facial expression, yawing, and head orientation are measured without attaching any additional equipment. Consequently, behavioral feature analysis is a cost-effective and easy-to-use solution.

Notably, the integration of deep learning techniques has significantly enhanced signal and image processing tasks in real word problems [22]. Deep learning has also witnessed remarkable advancements in the field of object detection [23]. These advancements have revolutionized various industries, including autonomous vehicles [24], security systems [25], and healthcare [26]. In recent years, deep learning has spearheaded a revolution in drowsiness detection across all categories, encompassing behavioral, biological, and vehicle movement indicators. The substantial impact of deep learning on these critical aspects of drowsiness detection has been transformative [2]. This study also considers behavioral features to detect drowsiness. Below, the relevant literature on drowsiness detection using deep learning and computer vision is discussed.

The use of spatial and temporal features is predominant in existing studies that utilize the behavioral data of drivers. For example, the study [27] used spatiotemporal data to detect fatigue among drivers by analyzing facial features. The authors proposed a fusion-based system to detect yawns, head pose estimation, and detection of somnolence. Three datasets were used for analysis: YawDD, DEAP, and MiraclHB. YawDD and MiraclHB contain behavioral features, whereas the DEAP dataset analyzes human emotional states, psychological signals, and electroencephalography (EEG). The proposed model achieved recall and precision of 84% and 85%, respectively. Similarly, ref. [28] focused on early drowsiness detection using temporal features. Occlusion criteria were used that measure the distance between the centers of the pupil and the horizontal length of the eye. The researcher used a support vector machine (SVM) classifier on publicly available benchmark data and achieved 89% accuracy.

Along the same lines, ref. [29] proposed a novel approach with two streams—a spatial– temporal graph convolutional network. The method leverages both spatial and sequential features. The two-stream framework employed in the method captures spatial and temporal features as well as first-order and second-order information at the same time. The anticipated method was evaluated on the YawDD and NTHU-DDD datasets, achieving an impressive average accuracy of 93.4% and 92.7%, respectively, demonstrating the feasibility and effectiveness of the method.

Another study [30] considered the most significant temporal features to detect drowsiness precisely. A novel algorithm was proposed using linear SVM for classification and the Dlib library to extract facial features for experimental analysis. A rarest IMM face dataset was used that contained all images with the open eyes of drivers. Samples of individuals related to diverse civilizations, colors, and environments were added to make the dataset more challenging and realistic. Occlusion is applied on each incoming frame while preprocessing to overcome the chances of false prediction. After handling the occlusion situation, the proposed system achieved 94.2% accuracy with open eyes.

Along the same lines, ref. [31] used temporal and spatial face features to detect drowsiness. The UTA-RLDD dataset used for experimentation has 30 h of videos of 60 participants with three classes: alert, low vigilant, and drowsy. In this study, two different models were proposed. The first model is based on a long short-term memory (LSTM) architecture for temporal feature extraction, and the second uses CNN and LSTM for spatial feature extraction. The Dlib library containing linear SVM classifier was used for temporal features with 79.9% accuracy; however, due to the multiple feature matrix, the computational time was increased. For spatial features, the Softmax classifier was used to obtain more accurate results, i.e., 97.5% accuracy.

According to [32], information related to the mouth and eyes is needed to classify them. This information is essential to obtain fast results and detect drowsiness states in drivers. In this regard, the study adopted a MTCNN model for drowsiness detection. Two public datasets were combined and used in this study including the YawDD video dataset of various people from various ethnic backgrounds, and the NTHU-DDD dataset containing five different scenarios, and each frame was labeled as ‘fatigue’ or ‘not fatigue’. The Dlib algorithm was implemented to detect the face, mouth, and eye regions. The experiments were performed at constant frame rates to calculate fatigue. As a result, the model achieved 98.81% accuracy.

Moreover, ref. [33] aimed to extract spatial and temporal features to detect drowsiness among drivers. The researchers proposed a new deep learning framework to collect drowsiness information from the spatial-temporal domain. The publicly available NTHU-DDD dataset was used for experimentation involving different participants with and without eyeglasses and variant illuminations. Different experiments were performed initially where the proposed approach achieved 82.8% accuracy with 3DcGAN. Additional experiments showed improved performance with an 87.1% accuracy using 3DcGAN+TLABiLSTM and 91.2% accuracy using 3DcGAN+TLABiLSTM and refinement. Similarly, ref. [34] employed a combination of multitask convolutional neural network (MTCNN) for face recognition and Dlib for locating facial key points. For extracting fatigue feature vectors from the facial key points of each frame, a temporal feature sequence was constructed and fed into an LSTM network to obtain an ultimate fatigue feature value. The proposed model achieved an average accuracy of 88% and 90% for YawDD and self-built datasets, respectively.

Real-time driver drowsiness detection is a challenging task, and a few studies have endeavored to perform this task. For example, ref. [35], drivers’ vigilance status was studied on real-time data using deep learning. The authors used the Haar-cascade method and CNN with the UTA-RLDD dataset, and five-fold validation was applied at a rate of 8.4 frames per second. The dataset contains real states of active and drowsy faces, so the trained model was expected to be more accurate and realistic. The selected CNN has low complexity too. The added novelty in the work is the creation of a custom dataset containing 122 videos of 10 participants. Various tuning hyperparameters were applied to achieve significant accuracy. On batch sizes of 200 and 500 epochs, it showed the best accuracy. The experimental results showed a 96.8% accuracy.

The study [36] presented a real-time system to analyze consecutive video frames using information entropy to detect fatigue. An improved YOLOv3-tiny CNN was used to capture facial regions. A geometric area called the face feature triangle (FFT) using the Dlib toolkit, facial landmarks, and coordinates of the facial regions were used to capture the relevant facial information. By utilizing the FFT, a face feature vector (FFV) is created that encapsulates all the necessary information to determine the fatigue state of the drivers. The proposed algorithm achieved a detection speed of over 20 frames per second with an accuracy rate of 94.32%. Ref. [37] collected real-time data for drowsiness detection and performed various experiments to validate it. Two hundred and twenty-three subjects participated, and frames were labeled into four classes. For experiments, data from 10 participants containing 245 videos each with 5 min duration were taken and split into a three to one ratio for training and validation. Various experiments were performed where a maximum accuracy of 64% was achieved.

Detecting drowsiness with open eyes is a challenging task. Ref. [30] used computer vision to detect real-time drowsiness among drivers. The system used eye blink duration as a key indicator for the accident avoidance system. The proposed approach detects the open and closed states of the eyes based on the eye aspect ratio (EAR). Experimental analysis of the YawDD dataset demonstrated that the system achieved an accuracy of approximately 92.5%.

The authors created a curated dataset of 50 subjects, 30 males and 20 females, with varying illumination conditions in [38]. Four deep convolutional neural networks (DCNNs) named Xception, ResNet101, InceptionV4, and ResNext101 with feature pooling methods were applied to this dataset. Most experiments achieved a 90% accuracy, but these models require significant computational resources. A low-complexity MobileNetV2 CNN was trained to maximize efficiency to overcome this problem. Weibull-based ResNext101 and MobileNetV2 models achieved 93.80% and 90.50% accuracy, respectively. Experiments were also performed on the benchmark dataset NTHU-DDD with the proposed algorithm that achieved an 84.21% accuracy.

The study [19] proposed a reliable fatigue detection system built on the CNN model. The study also proposed a fusion method to measure multiple physical features. The fusion method includes Haarlike, 68-Landmarkmodels, PERCLOS, and the MAR ratio. The VGG16 convolutional neural network for fatigue feature learning and the single-shot multi-box detector algorithm were adopted for better speed and accuracy. The VGG16 model helps to overcome the problems of poor environment, lighting conditions, and drivers wearing glasses to provide flexibility. Using four states of mouth, the experiments showed 90% accuracy on NTHU-DDD and other datasets.

In [39], the RLDD dataset was presented with the videos of 60 participants, of which 51 were male, and 9 were female. There are 180 videos, each 10 min long, with three alerts for low vigilance and drowsy classes. The researchers used five-fold experiments at a 4:1 training–testing ratio. The model was trained on 7000 blink sequences with a learning rate of 0.000053. Initially, the LSTM network achieved 61.4% accuracy, then experiments on the HM-LSTM network showed a 4% increase in accuracy and achieved 65.2% accuracy. Experiments also indicated that the HM-LSTM network performed well compared to fully connected layers and human judgment.

Table 1 provides a comparative summary of the discussed research works. The existing literature on driver drowsiness using facial features indicates that there is still room for improvement in driver drowsiness detection to improve the robustness and reliability of these approaches. Behavioral drowsiness detection is a difficult task. There is a need to develop a more effective and robust drowsiness detection approach.

## 3. Proposed Methodology

**Figure 1:**
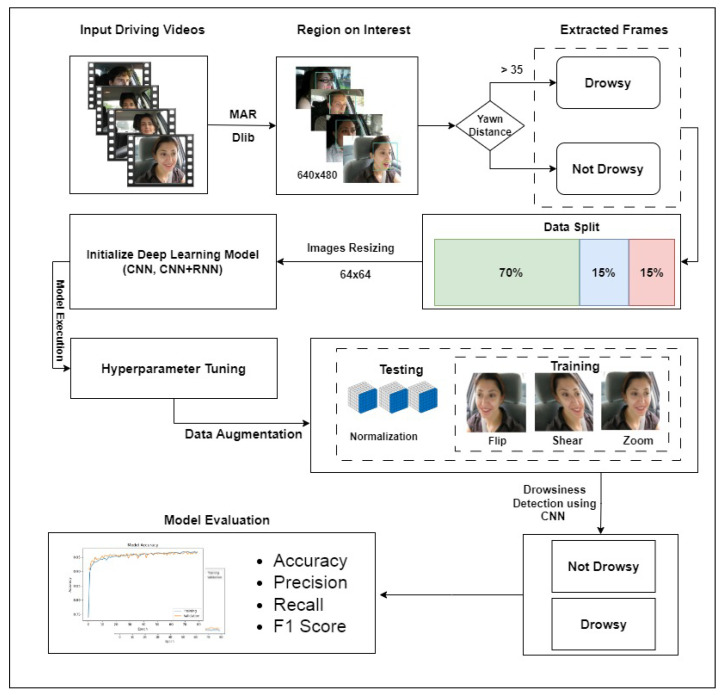
Architecture of proposed methodology.

This study proposes a deep neural network model for detecting drowsiness among drivers using images of behavioral features. The workflow diagram of the proposed approach is given in Figure 1. The process starts with video data acquisition for various people driving a vehicle. Videos contain normal driving behavior and yawning while driving. Region of interest is obtained from the videos for further classification of videos into yawning and no yawning. Data preparation and data preprocessing tasks are carried out. Various tasks are performed, such as resizing and cropping the video frames, normalizing the data, and extracting relevant features. Data are then split into training and test subsets for training and testing. This study builds and uses three deep neural networks for drowsiness detection among drivers. The performance of models is analyzed using accuracy, precision, recall, and F1 score. Further details of these tasks are provided in the subsequent sections.

### 3.1. Selected Dataset

To develop a drowsiness detection system, we used the YawDD dataset [41] that contains two sub-datasets named ‘Dash’ and ‘Mirror’ of drivers with various facial characteristics. The ‘Dash’ dataset has 13 female and 16 male participants. Table 2 shows the details of male and female participants for data collection.

These datasets contain videos of male and female drivers in different illumination conditions. Each participant has a video recorded from the car’s dashboard that contains scenes of driving, driving while talking, and driving while yawning. The ‘Mirror’ dataset contains 320 videos of 110 participants recorded from the mirror side of the car using a camera. Table 3 shows the details about the total number of videos for ‘Dash’ and ‘Mirror’ types for male and female participants.

**Figure 2:**
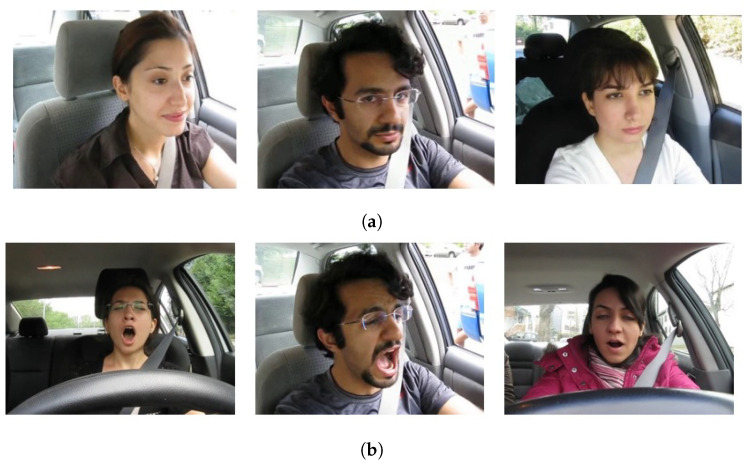
Samples from the YawDD video dataset, (**a**) Participants not yawning, and (**b**) Participants yawning.

There are three/four videos for each participant containing different facial conditions such as normal, talking or singing, and yawning. In both datasets, participants with and without glasses/sunglasses from different ethnicities are used for data collection. A few samples for the ‘yawning’ and ‘no_yawning’ classes are shown in Figure 2.

### 3.2. Segmentation and Classification

We classify or label to help distinguish between drowsy and not-drowsy images. The dataset is in the form of videos. It needs to be segmented and labeled to extract the area of interest and feed it to the proposed deep neural networks.

Before preprocessing, the video is converted into frames/images. The classification is performed based on the extraction of the region of interest. For this study, we have used a Dlib face detector library and 68 facial landmark predictors for the eyes and mouth [40,42]. The mouth region landmarks are extracted from the detected facial landmarks (“shape[48:68]”), which correspond to the points around the lips. With the help of these, we can calculate and detect features such as yawning and not yawning classes.

#### Mouth Aspect Ratio

The mouth aspect ratio (MAR) is the ratio of vertical length between the pair of lips to the horizontal length across the end edges of the lip. The distance between specific points on the upper and lower lips is calculated. These points are *mouth*[2], which is the top point on the lower lip, and *mouth*[10], which is the bottom point on the upper lip. The distance between these two points is referred to as the “yawn_distance”.

The yawn distance is calculated using the Euclidean distance formula between two specific points on the mouth region’s upper and lower lips. The formula to calculate the yawn distance is given as

(1) $$yawn\;distance=\sqrt{{\left(mouth\left[10\right]\left[0\right]-mouth\left[2\right]\left[0\right]\right)}^{2}+{\left(mouth\left[10\right]\left[1\right]-mouth\left[2\right]\left[1\right]\right)}^{2}}$$

where mouth[2][0] and mouth[2][1] correspond to the coordinates of the top point on the lower lip, and mouth[10][0] and mouth[10][1] correspond to the coordinates of the bottom point on the upper lip.

We set a threshold value of 35 to determine the yawn_distance. If the “yawn_distance” is less than or equal to the threshold, the frame is classified as not yawning, and the frame is saved in the “not_yawning” folder. If the calculated “yawn_distance” is greater than the threshold value, the frame is classified as yawning, and the frame is saved in the "yawning" folder. The used threshold is based on empirical observations on the dataset.

Based on segmentation, the output frames are stored in different relevant folders. The output images are in a .jpg file extension, each with a size of 640 × 480 pixels. The input videos are of different durations. The used algorithm converts video into images at 24 frames per second. The details of the output images/frames are given in Table 4.

### 3.3. Preprocessing

Before feeding the data to the neural network architecture, we perform further preprocessing. Various tasks like resizing and cropping the video frames, normalizing the data, and extracting relevant features are performed. The details of each step are provided in subsequent sections.

#### 3.3.1. Train Test Split

For training, testing, and validation purposes, the system takes the images from the input folders, splits them randomly, and stores them in the training, testing, and validation subfolders within the output folder according to the defined split ratios. The defined split ratio for our study is 70:15:15. The number of images after the train/test split is given in Table 5.

Batch size determines how many images are processed together in each training iteration. We have set the batch size to 32 for training and validation and 1 for testing. For testing, a batch size of 1 is often used to evaluate images individually.

#### 3.3.2. Setting Image Dimensions

We have set the input shape to (64, 64, 3), which means all images fed into neural network architectures are transformed to a 64-pixel width and 64-pixel height and have red, green, and blue (RGB) channels.

#### 3.3.3. Data Augmentation

**Figure 3:**
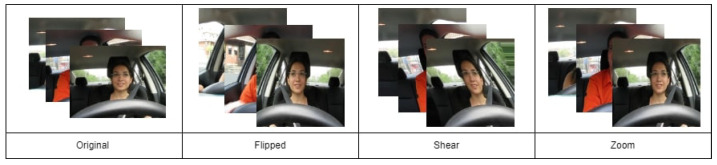
Samples from data augmentation.

In one of the experimental studies, we applied several data augmentation steps such as rescaling, flipping, zooming, and rotating, as suggested in earlier research [22,43], to address the issue of limited data availability, which causes over-fitting of the model. Below, an explanation of each data augmentation method is given along with its impact. Figure 3 shows the output of each type of augmentation applied in this study.

#### 3.3.4. Rescale (Normalization)

The input images are rescaled and set to 1/255 for their pixel values before being fed into the models. By dividing each pixel value by 255, the values of the pixels are normalized to the range of [0, 1]. This rescaling helps improve convergence during training [44].

#### 3.3.5. Shear Range

Shearing is a transformation that slants the image in a specified direction. The shear range parameter specifies the maximum angle in radians for the shearing transformation. It can help the model become more invariant to shear transformations that might occur in real-world scenarios. For the experiments, we set the shear range value to 0.2.

#### 3.3.6. Zoom Range

Zooming applies a scaling transformation to the image. The zoom range parameter specifies the range by which the image can be zoomed in or out. It can help the model learn to recognize objects at different scales and improve generalization. In this study, we set the zoom range value to 0.2.

#### 3.3.7. Horizontal Flip

This method horizontally flips the images with a 50% probability. It is often useful for tasks where the orientation of the object does not affect the classification result. We have also performed flipping. It is to be noted that the augmented images are not permanently stored. However, during training, different variations of each image will be created on the fly according to the augmentation parameters.

### 3.4. Proposed Deep Learning Architectures

Two deep neural network architectures are proposed in this study to process behavioral features. The first architecture is a CNN, and the second is a hybrid neural network combining CNN and RNN models.

#### 3.4.1. Deep CNN-1 Architecture

**Figure 4:**
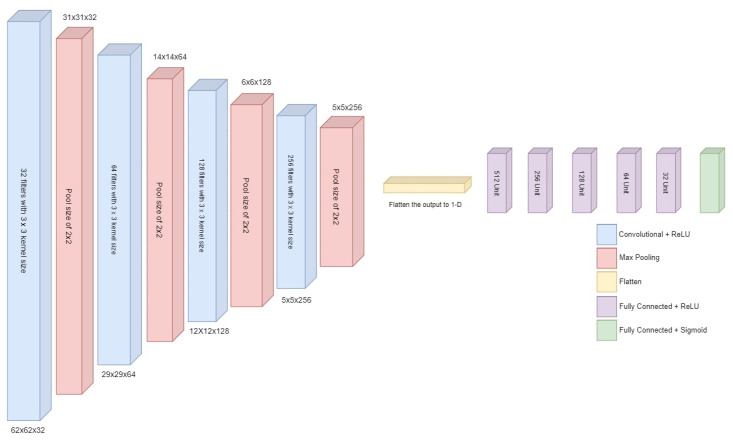
Architecture of proposed deep CNN.

The proposed deep neural network model takes input with the specified shape and generates a single probability output. The model starts with convolutional layers and max pooling layers to reduce spatial dimensions. The last part of the framework consists of fully connected layers (dense layers) leading to the output layer with a sigmoid activation function. An overview of the proposed deep neural network architecture is given in Figure 4.

The model is structured sequentially, with each layer added sequentially. It begins with a Conv2D layer consisting of 32 filters, each with a 3 × 3 kernel size. The activation function used in this layer is a rectified linear unit (ReLU). The input shape of this layer is specified using the ’input_shape’ parameter.

Following the Conv2D layer, a MaxPooling2D layer is added with a pool size of 2 × 2. This layer performs spatial downsampling, reducing the dimensions of the input. The model then incorporates three more sets of Conv2D and MaxPooling2D layers. The number of filters is increased to 64, 128, and 256, respectively, while maintaining the same 2 × 2 pool size. These additional layers aim to extract more intricate features from the input data. The subsequent flatten layer transforms the output from the previous layers into a 1-dimensional vector. This step is necessary to connect the convolutional layers to the dense layers.

The model then employs a series of dense layers, gradually reducing the number of units from 512 to 32. Each dense layer uses the ReLU activation function and processes the flattened features. Lastly, the output layer is added, consisting of two units with the ‘sigmoid’ activation function for binary classification according to the specified problem for this research.

Architectural details of the proposed CNN-1 are provided in Table 6. This model begins with the initial Conv2D layer, which produces an output with dimensions (None, 62, 62, 32) and has 896 parameters. This is followed by a MaxPooling2D layer, which reduces the spatial dimensions of the feature maps to (None, 31, 31, 32) with no additional parameters. The subsequent layers continue this pattern of alternating Conv2D and MaxPooling2D layers, progressively reducing the spatial dimensions and increasing the number of feature maps. The network then transitions to fully connected layers, starting with a flatten layer that transforms the 2D feature maps into a 1D vector with 1024 elements. The network further processes this vector through several dense layers, each with a decreasing number of units, culminating in a final dense layer with one unit, which is typically used for binary classification tasks. In total, this model has 1,087,809 parameters, all of which are trainable.

#### 3.4.2. Deep CNN-2 Architecture

**Figure 5:**
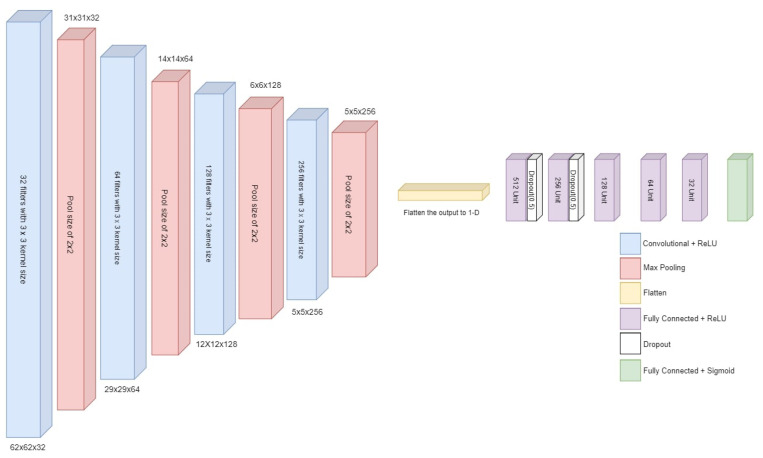
Architecture of proposed deep CNN-2.

This CNN model is designed for binary classification and is constructed as a sequential model, ensuring that data flows sequentially through the network. This is the foundation for stacking various layers together to create a functional neural network. The core of the model comprises four convolutional layers. These layers are pivotal in feature extraction from the input data. A small 3 × 3 filter is employed to scan the input images and create feature maps. The numbers 32, 64, 128, and 256 indicate the depth of these feature maps. As the depth increases, the model can learn increasingly complex features. The ‘relu’ activation function is employed in each of these layers. This function introduces non-linearity by outputting the input if it is positive, and zero otherwise. An overview of the proposed deep neural network architecture is given in Figure 5.

After each convolutional layer, there is a max-pooling layer. These layers reduce the spatial dimensions of the feature maps by selecting the maximum value from small regions (2 × 2). This downsampling helps in simplifying the data and retaining essential information. Following the convolutional layers, the flatten layer is employed. Its primary role is to transform the 2D feature maps into a 1D vector. This step is crucial as it readies the data for the transition from convolutional layers to fully connected layers.

The subsequent fully connected layers (1–5) are fundamental in leveraging the high-level feature representation produced by the convolutional layers. These layers are similar to traditional neural network layers, where each neuron is connected to every neuron in the previous and subsequent layers. The numbers 512, 256, 128, 64, and 32 represent the number of neurons in each layer. Larger numbers signify a more substantial capacity for learning intricate patterns. To introduce non-linearity, the ’relu’ activation function is used in these layers. Moreover, the addition of dropout layers after the first two fully connected layers (with a dropout rate of 0.5) serves as a regularization technique. During training, these layers randomly deactivate a fraction of the input units, preventing overfitting and enhancing generalization.

Finally, the output layer concludes the model. In this instance, it is comprised of a single neuron utilizing the ‘sigmoid’ activation function. The ‘sigmoid’ function transforms the output to a value between 0 and 1, making it suitable for estimating probabilities. This final output value represents the probability of the input data belonging to the positive class.

Table 7 provides the details of layers and the structure of each layer for the CNN-2 model. This model comprises a series of convolutional layers interleaved with activation functions, max-pooling layers, and fully connected layers. The convolutional layers, represented by “conv2d”, perform feature extraction from the input data, gradually reducing the spatial dimensions while increasing the number of feature maps. Following each convolutional layer, there is an “activation” layer, which typically applies a non-linear function to introduce non-linearity into the model. Max-pooling layers, denoted as “max_pooling2d”, further reduce the spatial dimensions of the feature maps by downsampling.

The model then transitions to a fully connected architecture with dense layers, marked as “dense”, which are crucial for making classification decisions. Activation layers follow these dense layers, adding non-linearity to the network. Dropout layers, such as “dropout” and “dropout_1”, help prevent overfitting by randomly deactivating a fraction of neurons during training. The final dense layer, “dense_5”, has a single unit, which is typical for binary classification tasks. It is important to note that the “activation_9” layer follows the output layer, but its purpose may vary based on the activation function used.

In total, the model has 1,087,809 parameters, all of which are trainable. These parameters are adjusted during the training process to learn and represent features in the data. The presence of dropout layers helps in mitigating overfitting.

#### 3.4.3. Hybrid CNN-RNN Architecture

**Figure 6:**
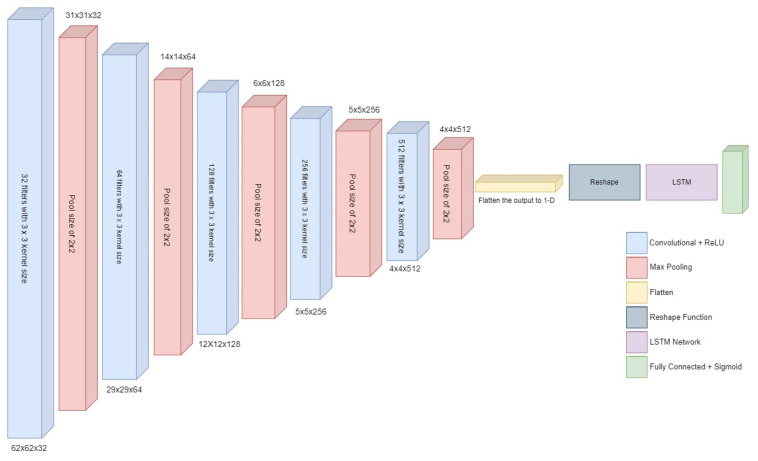
Architecture of hybrid deep CNN-RNN model.

A deep CNN-RNN hybrid model combines the capabilities of CNN and RNN to handle sequential data and leverage spatial and temporal relationships. To capture temporal information in the data, hybrid models include recurrent layers such as LSTM and convolutional layers [31]. As a result, they are compatible with applications that need sequential data, such as video analysis and speech recognition. The CNN component extracts spatial characteristics, while the RNN component represents temporal dynamics. The hybrid model combines the strengths of CNN and RNN models to handle both spatial and temporal information in the input data. Figure 6 provides an overview of the architecture and data flow in the hybrid deep CNN-RNN model.

The model begins by initializing a sequential model. Then, CNN layers are added to extract spatial features from the input data. These layers consist of Conv2D and MaxPooling2D layers, which apply filters to capture spatial patterns and downsample the input. After the CNN layers, a flatten layer is introduced to flatten the output from the CNN layers into a 1-dimensional vector. This step allows the data to be processed by the subsequent RNN layers. Following the flatten layer, a reshape layer is added. Its purpose is to reshape the output of the CNN layers, adding a time step dimension in preparation for the RNN layers. This reshaping transforms the output into a shape of (1, −1), where the first dimension represents the time step and the second dimension represents the flattened vector from the CNN layers.

Next, RNN layers are incorporated into the model. Specifically, an LSTM layer is used. LSTMs are a type of RNN that excels at capturing temporal dependencies in sequential data. This layer allows the model to learn patterns and relationships over time. Finally, an output layer with two units and a ’sigmoid’ activation function is added. This layer produces the final prediction, a probability value between 0 and 1. It indicates the likelihood of the input belonging to the positive class in a binary classification problem.

Table 8 provides a detailed architecture of the hybrid model. This neural network architecture is a deep and complex model. It starts with a series of convolutional layers, where the initial Conv2D layer processes input data into feature maps with dimensions (None, 62, 62, 32) and is accompanied by 896 parameters. Subsequent MaxPooling2D layers reduce the spatial dimensions of the feature maps. This pattern continues through multiple Conv2D and MaxPooling2D layers, resulting in a progressive reduction in spatial dimensions and an increase in the number of feature maps. The model takes an interesting turn at layer 9, introducing another Conv2D layer followed by a MaxPooling2D layer. However, in layer 10, “max_pooling2d_4”, there is a notable discrepancy where the spatial dimensions remain the same (4, 4, 512), which might be a data formatting or architectural choice.

The flatten layer (layer 11) transforms the 2D feature maps into a 1D vector with 8192 elements, setting the stage for subsequent layers. Layer 12, “reshape”, reconfigures the data into a 3D shape (None, 1, 8192). The model then incorporates an LSTM layer (layer 13), which is a type of recurrent layer designed for sequential data processing. This LSTM layer has a very high number of parameters, amounting to 4,260,352.

Finally, the model concludes with a dense layer (layer 14) with a single unit, typically used for binary classification tasks. The total number of parameters in this model is 5,009,857, all of which are trainable.

For all models, we defined the batch size as 32 for training and validation, specified the class mode to binary for classification, and set the number of epochs to 80 for training the model.

We have three distinct architectures with varying numbers of parameters and capabilities, as shown in Table 9. The first two models, labeled as CNN-1 and CNN-2, both belong to the CNN category. These models share identical total parameters, with a count of 1,087,809, all of which are trainable, meaning they can adapt to learn features from the data. Notably, they do not possess any non-trainable parameters.

In contrast, the third model, called “Hybrid CNN+RNN”, is a more complex architecture tailored for tasks involving sequential data or time series analysis. It boasts a substantially higher total parameter count of 5,009,857, again with all parameters being trainable. This model incorporates a convolutional component for feature extraction, followed by recurrent layers, specifically LSTM units, for sequence analysis. The absence of non-trainable parameters implies that the entire architecture is adaptable to the learning process.

## 4. Results and Discussions

Six experiments are conducted on the complete dataset. We have proposed three deep neural network architectures for training and analysis. Each model is trained with and without data augmentation. The design of a deep learning model for drowsiness detection is based on several key factors and considerations, primarily to ensure its accuracy, efficiency, and real-world applicability. As we have the data in the form of frames of videos and we have binary class problems, that is the reason we develop these model architectures.

The findings of each experiment are discussed in this section. These experiments aim to achieve maximum classification accuracy and optimized performance for other quantitative measures for drowsiness detection among drivers. To measure the effect and performance of deep learning architecture in predicting yawning among drivers, the performance is assessed using standard metrics like accuracy, confusion matrix, precision, recall, and F1 score. We have used these measures to compare our solution with the relevant existing literature.

### 4.1. Experiments with Deep CNN Architecture

**Figure 7:**
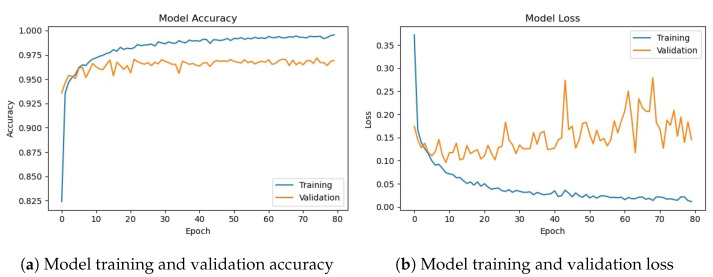
Training/validation model accuracy and loss graph without data augmentation.

The deep CNN base architecture model is used in the experiments using training and validation data. The model accuracy and loss graphs for the proposed CNN model without data augmentation are shown in Figure 7. It can be observed that the model starts with zero training accuracy; however, it improves as the number of epochs proceeds.

**Figure 8:**
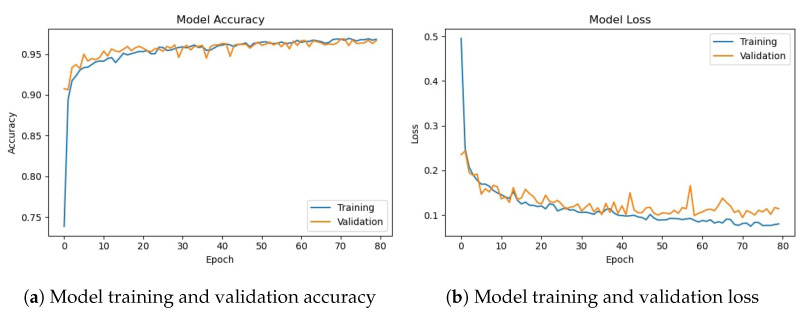
Training/validation model accuracy and loss graph with data augmentation.

Figure 8 shows training and validation accuracy and loss for the CNN-1 model using the augmented data. Results show that model training and validation accuracy show a very similar trend as the number of epochs increases, contrary to the graphs on the original data where the model training and validation accuracy and loss have different trends.

Table 10 shows the training and validation of the proposed CNN-1 model without and with data augmentation. In the first experiment without data augmentation, the proposed model achieved a 96.34% accuracy rate for testing and 99.69% in training, respectively. Similarly, in the second experiment with data augmentation, the proposed architecture achieved a 95.99% accuracy rate for testing data and 96.85% for training, respectively. Experimental results show that the proposed architecture without data augmentation has achieved the highest accuracy.

The performance evaluation matrix, including precision, recall, and F1 score for both classes of the dataset, are shown in Table 11. The precision score by the proposed CNN model without data augmentation is 0.9728 for the drivers belonging to the ‘not_yawning’ class and 0.9480 for the drivers belonging to the ‘yawning’ class indicating that 97.28% of the instances predicted as ‘Not Yawning’ are actually ‘Not Yawning’ and 94.80% of the instances predicted as ‘Yawning’ are actually ‘Yawning’.

Similarly, the recall for the ‘Not Yawning’ class is 0.9680. For the ‘Yawning’ class, it is 0.9558, indicating 96.80% and 95.58% of instances were correctly predicted as ‘Not Yawning’ and ‘Yawning’, respectively. The F1 score is considered more reliable, especially for scenarios where the class distribution is imbalanced, as it combines both precision and recall. The F1 score is 0.9704 (97.04%) for 1409 instances for the ‘not_yawning’ class and 0.9519 (95.19%) for 860 instances for the ‘yawning’ class, respectively. It shows that there is a significant balance between precision and recall.

For experiments involving data augmentation, the results of the CNN-1 model are slightly different. The precision score by the CNN-1 model is 0.9551 for the ‘not_yawning’ class and 0.9683 for the ‘yawning’ class, which indicates that 95.51% of the instances predicted as ‘Not Yawning’ are actually ‘Not Yawning’ and 96.83% of the instances predicted as ‘Yawning’ are actually ‘Yawning’. Similarly, the recall for the ‘Not Yawning’ class is 0.9815. For the ‘Yawning’ class, it is 0.9244, indicating 98.15% and 92.% instances are correctly predicted as ‘Not Yawning’ and ‘Yawning’, respectively. The F1 score is 0.9682 (96.82%) for 1409 instances for the ‘not_yawning’ class and 0.9459 (95.59%) for 860 instances for the ‘yawning’ class, respectively; it shows that there is a significant balance between precision and recall.

**Figure 9:**
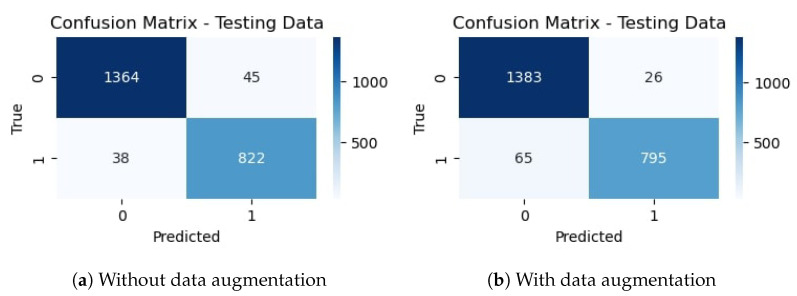
Confusion matrix of proposed CNN without and with data augmentation.

The confusion matrix for the proposed CNN-1 model without data augmentation is given in Figure 9a. It indicates that out of 2269 testing images, 2186 are classified correctly by the proposed CNN model. It means the accuracy rate for the model is 96.34%. Similarly, Figure 9b shows the confusion matrix for the proposed architecture with data augmentation, which indicates that out of 2269 testing images, 2178 are correctly classified. It means that the accuracy rate for the model is 95.99%.

### 4.2. Experiments with Deep CNN-2 Architecture

**Figure 10:**
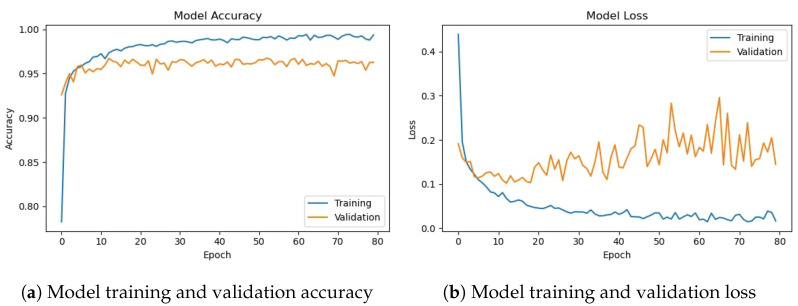
Training/validation model accuracy and loss graph of CNN-2 model without data augmentation.

The deep CNN-2 base architecture model is used in the experiments using training and validation data. The model accuracy and loss graphs for the proposed CNN-2 model without data augmentation are shown in Figure 10. Similar to the previous model, it starts with zero training accuracy, but improves as the number of epochs proceeds.

**Figure 11:**
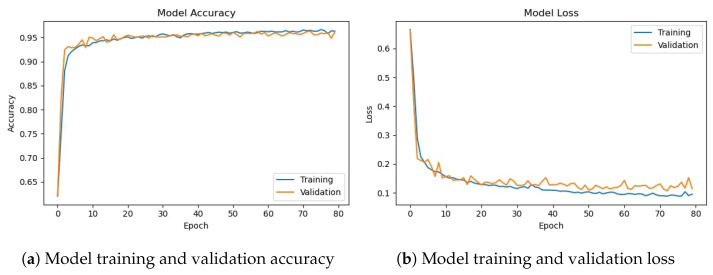
Training/validation model accuracy and loss graph of CNN-2 model with data augmentation.

Figure 11 shows training and validation accuracy and loss for the CNN-2 model using the augmented data. Similar to CNN-1, CNN-2 model training and validation accuracy shows a very similar trend as the number of epochs increases, contrary to the graphs on the original data where the model training and validation accuracy and loss have different trends.

Table 12 shows the training and validation of the proposed CNN-2 model without and with data augmentation. In the first experiment without data augmentation, the proposed CNN-2 model achieved a 96.69% accuracy rate for testing and 99.41% in training, respectively. Similarly, in the second experiment with data augmentation, the proposed CNN-2 architecture achieved a 95.50% accuracy rate for testing data and 95.94% for training, respectively. Experimental results show that the proposed architecture without data augmentation has achieved the highest accuracy.

The performance evaluation matrix, including precision, recall, and F1 score for both classes of the dataset, are shown in Table 13. The precision score by the proposed CNN-2 model without data augmentation is 0.9730 for the drivers belonging to the ‘not_yawning’ class and 0.9569 for the drivers belonging to the ‘yawning’ class indicating that 97.30% of the instances predicted as ‘Not Yawning’ are actually ‘Not Yawning’ and 95.69% of the instances predicted as ‘Yawning’ are actually ‘Yawning’. Similarly, the recall for the ‘Not Yawning’ class is 0.9737. For the ‘Yawning’ class, it is 0. 9558, indicating 97.37% and 95.58% instances were correctly predicted as ‘Not Yawning’ and ‘Yawning’, respectively. The F1 score is 0.9733 (97.33%) for 1409 instances for the ‘not_yawning’ class and 0.9563 (95.63%) for 860 instances for the ‘yawning’ class, respectively. It shows that there is a significant balance between precision and recall.

For experiments involving data augmentation, the results of the CNN-2 model are slightly different. The precision score by the CNN-2 model is 0.9516 for the ‘not_yawning’ class and 0.9610 for the ‘yawning’ class, which indicates that 95.16% of the instances predicted as ‘Not Yawning’ are actually ‘Not Yawning’ and 96.10% of the instances predicted as ‘Yawning’ are actually ‘Yawning’. Similarly, the recall for the ‘Not Yawning’ class is 0.9772. For the ‘Yawning’ class, it is 0.9186, indicating 97.72% and 91.86% instances are correctly predicted as ‘Not Yawning’ and ‘Yawning’, respectively. The F1 score is 0.9642 (96.42%) for 1409 instances for the ‘not_yawning’ class and 0.9393 (93.93%) for 860 instances for the ‘yawning’ class, respectively; it shows that there is a significant balance between precision and recall.

**Figure 12:**
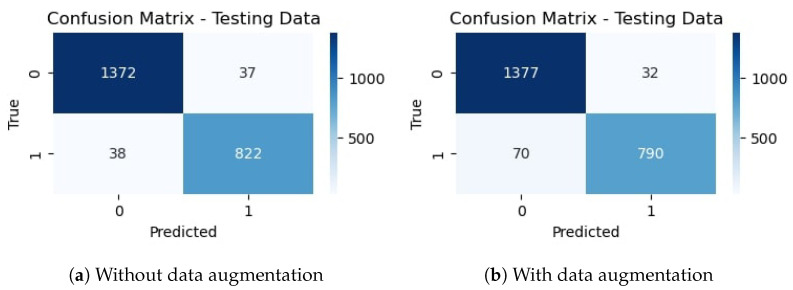
Confusion matrix of proposed CNN-2 without and with data augmentation.

The confusion matrix for the proposed CNN-2 model without data augmentation is given in Figure 12a. It indicates that out of 2269 testing images, 2194 are classified correctly by the proposed CNN-2 model. It means the accuracy rate for the model is 96.69%. Similarly, Figure 12b shows the confusion matrix for the proposed architecture with data augmentation, which indicates that out of 2269 testing images, 2167 are correctly classified. It means that the accuracy rate for the model is 95.50%.

### 4.3. Experiments with Deep CNN-RNN Architecture

**Figure 13:**
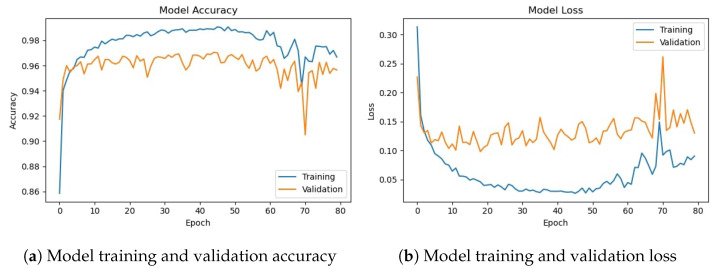
Training/validation model accuracy and loss graph of hybrid CNN-RNN model without data augmentation.

In the third and fourth experiments, we implement hybrid deep CNN-RNN architecture for driver drowsiness detection. The model accuracy and loss graphs of the hybrid model without data augmentation are shown in Figure 13. It is observed that the model starts poorly with training accuracy, but improves as the number of epochs proceeds. The best accuracy is obtained with 47 epochs, but after that, the training and validation accuracy start reducing.

**Figure 14:**
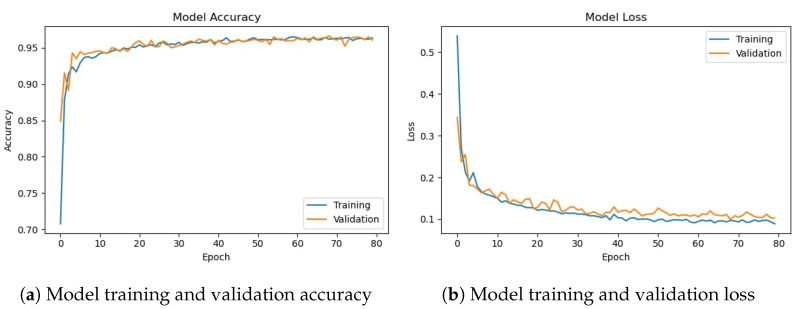
Training/validation model accuracy and loss graph of hybrid CNN-RNN model with data augmentation.

Figure 14 shows training and validation accuracy and loss of hybrid CNN-RNN with data augmentation. It can be seen that contrary to the behavior of the model on the original data where training and validation curves have different trends, model training and validation curves show very similar trends as the number of epochs grow.

Table 14 shows the training and validation accuracy of the hybrid CNN-RNN model using data augmentation and no augmentation. The proposed hybrid model obtained a 95.24% accuracy without augmentation and 97.55% training accuracy. On the other hand, with data augmentation, it achieved 95.64% and 96.28% accuracy for testing and training, respectively. Results show that results are better if the model is trained on the original dataset without data augmentation.

Results regarding precision, recall, and F1 score are given in Table 15. The precision score for the proposed CNN-RNN model without data augmentation is 0.9514 for the ‘not_yawning’ class and 0.9541 for the ‘yawning’ class, indicating that 95.14% of the instances predicted as ‘Not Yawning’ are actually ‘Not Yawning’ and 95.41% of the instances predicted as ‘Yawning’ are actually ‘Yawning’. Similarly, the recall for the ‘Not Yawning’ and ‘Yawning’ classes is 0.9730 and 0.9186, respectively, indicating 97.30% and 91.86% instances are correctly predicted. The F1 score is 0.9621 (96.21%) for 1409 instances for the ‘not_yawning’ class and 0.9360 (93.60%) for 860 instances for the ‘yawning’ class, respectively, showing that the model does not have overfitting.

The precision score for the CNN-RNN model using augmented data is 0.9517 for the ‘not_yawning’ class and 0.9646 for the ‘yawning’ class. Results indicate that 95.17% and 96.46% of the ‘Not Yawning’ and ‘Yawning’ classes are predicted correctly. In the same way, recall scores of 0.9794 and 0.9186 for not yawning and yawning indicate superior results. F1 scores of 0.9654 (96.54%) for 1409 instances of the ‘not_yawning’ class and 0.9410 (95.10%) for 860 instances of the ‘yawning’ class, respectively, also show a balance between precision and recall.

**Figure 15:**
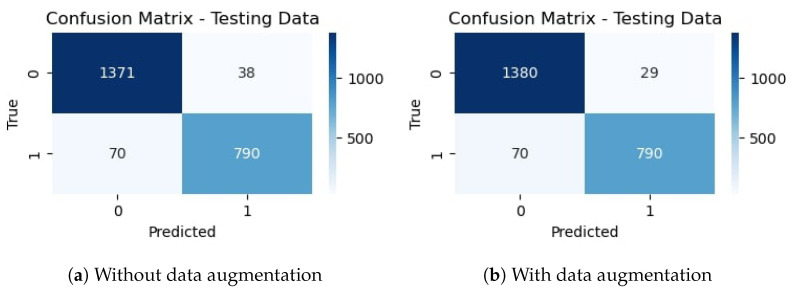
Confusion matrix of hybrid CNN-RNN without and with data augmentation.

The confusion matrix for the proposed CNN model without data augmentation in Figure 15a indicates that out of 2269 testing images, 2161 are classified correctly by the hybrid CNN-RNN model. It indicates that the accuracy rate for the model is 95.24%. Similarly, Figure 15b shows the confusion matrix for the proposed architecture with data augmentation, which indicates that out of 2269 testing images, 2170 are correctly classified showing an accuracy rate for the model is 95.64%.

### 4.4. Computational Time of Models

We set the batch size to 32 for training and the number of epochs to 80 and trained the models on the YawDD dataset. Table 16 provides the training time of all models employed in this study.

Firstly, two variants of CNN were considered. CNN-1, without data augmentation, exhibited a training and testing time of 3.24 h. When data augmentation techniques were applied to CNN-1, the time increased slightly to 3.70 h. On the other hand, CNN-2, which is another variant of the CNN architecture, required 2.89 h for training and testing without data augmentation and 3.01 h when data augmentation was incorporated. These results demonstrate that CNN-2 was generally more time-efficient compared to CNN-1, while data augmentation increased training time for both architectures.

Additionally, a hybrid CNN-RNN architecture was assessed, again with and without data augmentation. Without data augmentation, this architecture demanded 3.74 h for training and testing, making it one of the most time-consuming options in this study. The introduction of data augmentation increased the time marginally to 3.82 h.

### 4.5. Comparison with Existing Studies

This study primarily focuses on the effectiveness of our model in detecting drowsiness indicators within the scope of the provided dataset. The performance of the proposed CNN model is compared with relevant studies available in the literature for the detection of drowsiness among drivers. Table 17 shows the performance comparison results. This study used the YawDD dataset for experimental analysis, so we are considering only those existing studies for comparison that have used the YawDD dataset for experiments. Based on classification accuracy, the proposed CNN model outperforms existing studies on driver drowsiness detection.

To capture facial regions in complex driving conditions, [36] used improved YOLOv3-tiny CNN. The proposed algorithm in this study achieved an accuracy rate of 94.32% at a detection speed of over 20 frames per second. In [34], the authors employed a combination of MTCNN for face detection and Dlib for locating facial key points. The proposed model achieves an 88% average accuracy. A real-time fatigue detection system has been developed that calculates eye blink duration as a key indicator for accident avoidance systems. The experimental analysis on the YawDD dataset shows that it achieves an accuracy of 92.5% [30]. Lastly, [29] proposed an approach using two streams of spatial-temporal graph convolutional networks for driver drowsiness detection. The model leverages spatial and temporal features and achieved 93.4% accuracy on the YawDD dataset.

### 4.6. Discussion

This study proposes a deep CNN model for accurately detecting driver drowsiness from videos. In addition, a hybrid model comprising CNN and RNN is also designed for performance comparison. Drivers’ behavioral features are utilized to train and test the models. Moreover, to resolve the data imbalance problem, data augmentation is also utilized. A summary of results employing both models and data augmentation is presented in Table 18. It shows that the average accuracy for both models is very close. The CNN model achieves an average accuracy of 98.01% without data augmentation, which is higher than the average accuracy of the hybrid CNN-RNN model for all experiments.

Experimental results indicate that in general, the results of deep CNN, which only considers spatial features, perform slightly better than the hybrid deep CNN-RNN model, which considers spatial-temporal features. Performance measures like precision, recall, and F1 score are also considered to compare the results. Overall, both models achieve amazing performance with and without data augmentation, showing a very close difference. Although we achieve better accuracy without data augmentation, it is a powerful technique to enhance model performance and generalization ability. When we look at the training validation graphs, we can conclude that after data augmentation, the accuracy might be reduced, but the generalization of the models is enhanced.

While this research has demonstrated the effectiveness of the deep learning model for drowsiness detection, it is essential to acknowledge certain limitations that are associated with the use of DLib and the potential impacts of these limitations in real-world scenarios [45]. In real-world scenarios, it is challenging to ensure that a driver maintains a consistent head orientation toward the installed camera. DLib, like many other vision-based methods, primarily focuses on a single point of monitoring, typically the driver’s face. This approach does not consider the broader context of the driver’s behavior, which may include fatigue-related cues from other parts of the body (e.g., body posture or hand movements).

Drowsiness among drivers causes injuries and deaths of millions of people annually, so there is a need to develop a system with high accuracy, precision, and recall. Detection of drowsiness is the key factor for successfully preventing road accidents. This study focused on drowsiness detection among drivers using deep learning architectures. We try to develop a more accurate model for predicting yawning or drowsiness among drivers. The results show that the proposed architectures, which only consider spatial features, perform better without data augmentation on the selected dataset. Overall, all three models show exceptional performance with and without data augmentation, showing a very close difference.

Existing research works on drowsiness detection provide various deep learning architectures and report results regarding accuracy, sensitivity, etc. This study makes a difference in the following context:

i Behavioral feature-based drowsiness detection: While drowsiness detection using facial features is a known area of research, our study specifically focuses on using behavioral features, such as yawning, as a means to detect drowsiness. This approach offers a novel perspective on addressing drowsiness detection, which complements existing methods.
ii Three deep learning architectures: We propose and compare two deep learning architectures, a deep CNN and a hybrid CNN-RNN, for drowsiness detection. Using a hybrid architecture that considers spatial and temporal features is innovative and can potentially lead to more robust results, especially in dynamic scenarios like drowsy driving.
iii Data augmentation: Our study highlights the impact of data augmentation techniques on model performance. This analysis, coupled with the focus on behavioral features, contributes to the novelty of our research. It demonstrates that data augmentation is a valuable tool in enhancing the generalization of models in this context.
iv Comparison with existing studies: Our research includes a comparison with existing studies, specifically on the same YawDD dataset. By achieving better accuracy compared to these existing studies, our research showcases a novel and effective approach to drowsiness detection among drivers.

Overall, the novelty of the study lies in the unique combination of using behavioral features, proposing three deep learning architectures, analyzing the impact of data augmentation, and achieving higher accuracy compared to existing studies, all within the context of addressing drowsiness detection among drivers.

## 5. Conclusions and Future Work

Drowsiness among drivers is one of the major causes of road accidents, which can lead to severe injuries and deaths. It is essential to detect drowsiness among drivers using low-cost, highly effective methods. For that purpose, this research develops a drowsiness detection model using behavioral features such as changes in the eyes and mouth during drowsiness. A 15-layer custom CNN model is proposed for driver drowsiness detection with high accuracy and robustness. The YawDD benchmark dataset is used for experimental analysis. The dataset was preprocessed involving segmentation and classification of the video streams into frames for the ’Not Yawning’ and ’Yawning’ classes. The Dlib with 68 facial landmark predictors is used to detect areas of interest including eyes and mouth. The proposed CNN model is tested with and without data augmentation to detect drowsiness. Experimental results demonstrate that the proposed model achieved an average accuracy of 96.69% without data augmentation, which is superior to existing models on drowsiness detection. Results depict that using data augmentation marginally reduces the detection accuracy of the model but improves its robustness and generalization. This study utilizes only a single dataset, YawDD, indicating a lack of generalization on other datasets. In the future, we intend to test the model on additional datasets. Moreover, more facial features such as the eyes, nose, head, and ear can be considered for driver drowsiness detection.

## Figures and Tables

**Table 1:** Comparative summary of discussed research works.

Ref.	Base Parameters	Model	Accuracy (Test)	Dataset (s)
[27]	Facial Regions	Fusion System (Analysis of Mixed Datasets)	93.38%, 96.68%	YawDD, DEAP, MiraclHB
[28]	Facial Regions	SVM+LSTM	89%	UTA-RLDD
[19]	Eyes and Mouth	Multi-physical Feature Fusion Detection Method Detection Method based on Deep Learning SSD+VGG16	95.7%, 91.4% (Custom), 91.88%	(Public) Homemade Dataset, NTHU-DDD
[38]	Facial Regions	Weibull-based MobileNetV2 Weibull-based ResNext101 MTCNN+Weibull Pooling+ResNext101	93.8%, 90.5%, 84.21%	Custom Dataset (Total 50, 30 male, 20 female) NTHU-DDD
[29]	Facial Regions	2-stream spatial-temporal graph convolutional network (2s-STGCN)	93.4%, 92.7%	YawDD, NTHU-DDD
[35]	Facial Regions	3D Deep CNN CNN (LeNet)	96.80%	UTA-RLDD Custom Dataset (10 Subjects)
[30]	Facial Regions	Linear Support Vector Machine (SVM) as classifier + Dlib	92.5%	YawDD
[40]	Facial Regions	SVM + Dlib facial feature predictor	94.55%	IMM face Dataset + Other Mixed Samples
[31]	Facial Regions	TFBI LSTM, CNN-LSTM	79.9% Temporal, 97.5% Spatial	UTA-RLDD
[34]	Mouth and Eyes	MTCNN+DLIB+LSTM NN	88%, 90%	YawDD Self-Built Dataset
[32]	Eyes and Mouth	SVM and Adaboost + Multitask ConNN	98.81%	YawDD and NthuDDD
[36]	Facial Regions	YOLOv3-tiny CNN + Face Feature, Triangle (FFT) + Face Feature Vector (FFV)	94.32%	YawDD
[37]	Facial Regions	Conv2D-raw + SMOTE	64%	Real-Time Generated Dataset
[33]	Facial Regions	3DcGAN+TLABiLSTM+Refinement, 3DcGAN+TLABiLSTM, 3DcGAN	91.20%, 87.1%, 82.8%	NthuDDD
[39]	Eyes	HM-LSTM network, LSTM network	65.2%, 61.4%	Custom Dataset

**Table 2:** Total number of participants in the dataset.

Gender	Dash	Mirror	Total
Female	13	43	56
Male	16	47	63
Overall			119

**Table 3:** Total number of videos in the dataset.

Gender	Dash	Mirror	Total
Female	13	156	169
Male	16	164	180
Overall			349

**Table 4:** Total number of frames for the YawDD dataset.

Output Folder	Dash Frames	Mirror Frames	Total Frames
no_yawning	6567	2832	9399
yawning	3314	2423	5737

**Table 5:** Train/Test split for the YawDD dataset.

Class	Training	Validation	Testing
Yawning	4015	860	862
Not Yawning	6579	1409	1411

**Table 6:** Architecture of proposed CNN-1 model.

Layer (Type)	Output Shape	Parameters
conv2d (Conv2D)	(None, 62, 62, 32)	896
max_pooling2d (MaxPooling2D)	(None, 31, 31, 32)	0
conv2d_1 (Conv2D)	(None, 29, 29, 64)	18,496
max_pooling2d_1 (MaxPooling2D)	(None, 14, 14, 64)	0
conv2d_2 (Conv2D)	(None, 12, 12, 128)	73,856
max_pooling2d_2 (MaxPooling2D)	(None, 6, 6, 128)	0
conv2d_3 (Conv2D)	(None, 4, 4, 256)	295,168
max_pooling2d_3 (MaxPooling2D)	(None, 2, 2, 256)	0
flatten (Flatten)	(None, 1024)	0
dense (Dense)	(None, 512)	524,800
dense_1 (Dense)	(None, 256)	131,328
dense_2 (Dense)	(None, 128)	32,896
dense_3 (Dense)	(None, 64)	8256
dense_4 (Dense)	(None, 32)	2080
dense_5 (Dense)	(None, 1)	33

**Table 7:** Architecture of proposed CNN-2 model.

Layer (Type)	Output Shape	Parameters
conv2d (Conv2D)	(None, 62, 62, 32)	896
activation (Activation)	(None, 62, 62, 32)	0
max_pooling2d (MaxPooling2D)	(None, 31, 31, 32)	0
conv2d_1 (Conv2D)	(None, 29, 29, 64)	18,496
activation_1 (Activation)	(None, 29, 29, 64)	0
max_pooling2d_1 (MaxPooling2D)	(None, 14, 14, 64)	0
conv2d_2 (Conv2D)	(None, 12, 12, 128)	73,856
activation_2 (Activation)	(None, 12, 12, 128)	0
max_pooling2d_2 (MaxPooling2D)	(None, 6, 6, 128)	0
conv2d_3 (Conv2D)	(None, 4, 4, 256)	295,168
activation_3 (Activation)	(None, 4, 4, 256)	0
max_pooling2d_3 (MaxPooling2D)	(None, 2, 2, 256)	0
flatten (Flatten)	(None, 1024)	0
dense (Dense)	(None, 512)	524,800
activation_4 (Activation)	(None, 512)	0
dropout (Dropout)	(None, 512)	0
dense_1 (Dense)	(None, 256)	131,328
activation_5 (Activation)	(None, 256)	0
dropout_1 (Dropout)	(None, 256)	0
dense_2 (Dense)	(None, 128)	32,896
activation_6 (Activation)	(None, 128)	0
dense_3 (Dense)	(None, 64)	8256
activation_7 (Activation)	(None, 64)	0
dense_4 (Dense)	(None, 32)	2080
activation_8 (Activation)	(None, 32)	0
dense_5 (Dense)	(None, 1)	33
activation_9 (Activation)	(None, 1)	0

**Table 8:** Architecture of hybrid (CNN+RNN) model.

Layer (Type)	Output Shape	Parameters
conv2d (Conv2D)	(None, 62, 62, 32)	896
max_pooling2d (MaxPooling2D)	(None, 31, 31, 32)	0
conv2d_1 (Conv2D)	(None, 29, 29, 64)	18,496
max_pooling2d_1 (MaxPooling2D)	(None, 14, 14, 64)	0
conv2d_2 (Conv2D)	(None, 12, 12, 128)	73,856
max_pooling2d_2 (MaxPooling2D)	(None, 6, 6, 128)	0
conv2d_3 (Conv2D)	(None, 5, 5, 256)	131,328
max_pooling2d_3 (MaxPooling2D)	(None, 5, 5, 256)	0
conv2d_4 (Conv2D)	(None, 4, 4, 512)	524,800
max_pooling2d_4 (MaxPooling2D)	(None, 4, 4, 512)	0
flatten (Flatten)	(None, 8192)	0
reshape (Reshape)	(None, 1, 8192)	0
lstm (LSTM)	(None, 128)	4,260,352
dense (Dense)	(None, 1)	129

**Table 9:** Number of parameters for each architecture.

Model	Parameters	Trainable Parameters	Non-Trainable Parameters
CNN-1	1,087,809	1,087,809	0
CNN-2	1,087,809	1,087,809	0
Hybrid CNN+RNN	5,009,857	5,009,857	0

**Table 10:** Accuracy results for proposed CNN model.

Augmentation	Training Accuracy	Testing Accuracy
Yes	96.85%	95.99%
No	99.69%	96.34%

**Table 11:** Results of proposed CNN model.

Augmentation	Not Yawning (0)			Yawning (1)		
	Precision	Recall	F1-Score	Precision	Recall	F1-Score
No	0.9728	0.9680	0.9704	0.9480	0.9558	0.9519
Yes	0.9551	0.9815	0.9682	0.9683	0.9244	0.9459

**Table 12:** Accuracy result for proposed CNN-2 model.

Augmentation	Training Accuracy	Testing Accuracy
Yes	96.94%	95.50%
No	99.41%	96.69%

**Table 13:** Experimental results of proposed CNN-2 model for ‘yawning’ and ‘not yawning’.

Augmentation	Not Yawning (0)			Yawning (1)		
	Precision	Recall	F1-Score	Precision	Recall	F1-Score
No	0.9730	0.9737	0.9733	0.9569	0.9558	0.9563
Yes	0.9516	0.9772	0.9642	0.9610	0.9186	0.9393

**Table 14:** Accuracy result for hybrid CNN-RNN model.

Augmentation	Training Accuracy	Testing Accuracy
Yes	97.55%	95.25%
No	96.28%	95.64%

**Table 15:** Experimental results of hybrid CNN-RNN model.

Augmentation	Not Yawning (0)			Yawning (1)		
	Precision	Recall	F1-Score	Precision	Recall	F1-Score
No	0.9514	0.9730	0.9621	0.9541	0.9186	0.9360
Yes	0.9517	0.9794	0.9654	0.9646	0.9186	0.9410

**Table 16:** Comparison of computational time for each model.

Architecture	Total Time (in Hours)
CNN-1 without Data Augmentation	3.24
CNN-1 with Data Augmentation	3.70
CNN-2 without Data Augmentation	2.89
CNN-2 with Data Augmentation	3.01
Hybrid CNN-RNN without Data Augmentation	3.74
Hybrid CNN-RNN with Data Augmentation	3.82

**Table 17:** Proposed model’s comparison with other studies.

Reference	Year	Methodology	Accuracy
[36]	2020	YOLOv3-tiny CNN + Face Feature Triangle + Face Feature Vector	94.32%
[34]	2021	MTCNN+DLIB+LSTM NN	88%
[30]	2021	Dlib + linear Support Vector Machine	92.5%
[29]	2022	2s-STGCN	93.4%
This study	2023	Dlib+ 15 layers CNN without data augmentation	96.69%

**Table 18:** Overall performance of deep learning architectures.

Architecture	Training Accuracy	Testing Accuracy
CNN-1 without Data Augmentation	99.69%	96.34%
CNN-1 with Data Augmentation	96.85%	95.99%
CNN-2 without Data Augmentation	99.41%	96.69%
CNN-2 with Data Augmentation	95.94%	95.50%
Hybrid CNN-RNN without Data Augmentation	97.55%	95.24%
Hybrid CNN-RNN with Data Augmentation	96.28%	95.64%

## Data Availability

Not applicable.
